# Emerging roles of platelets in cancer biology and their potential as therapeutic targets

**DOI:** 10.3389/fonc.2022.939089

**Published:** 2022-07-22

**Authors:** Lei Wang, Xueying Wang, Erliang Guo, Xionghui Mao, Susheng Miao

**Affiliations:** ^1^ Department of Head and Neck Surgery, Harbin Medical University Cancer Hospital, Harbin, China; ^2^ Department of Otolaryngology Head and Neck Surgery, Xiangya Hospital, Central South University, Changsha, China; ^3^ Department of Surgery, The Second Affiliated Hospital of Harbin Medical University, Harbin, China

**Keywords:** platelet activation, platelet aggregation activity, immune escape, NETosis, tumor metastasis, liquid biopsy, tumor treatment

## Abstract

The main role of platelets is to control bleeding and repair vascular damage *via* thrombosis. They have also been implicated to promote tumor metastasis through platelet-tumor cell interactions. Platelet-tumor cell interactions promote tumor cell survival and dissemination in blood circulation. Tumor cells are known to induce platelet activation and alter platelet RNA profiles. Liquid biopsies based on tumor-educated platelet biomarkers can detect tumors and correlate with prognosis, personalized therapy, treatment monitoring, and recurrence prediction. Platelet-based strategies for cancer prevention and tumor-targeted therapy include developing drugs that target platelet receptors, interfere with the release of platelet particles, inhibit platelet-specific enzymes, and utilize platelet-derived “nano-platelets” as a targeted drug delivery platform for tumor therapy. This review elaborates on platelet-tumor cell interactions and the molecular mechanisms and discusses future research directions for platelet-based liquid biopsy techniques and platelet-targeted anti-tumor strategies.

## 1 Introduction

Mature megakaryocytes produce platelets, which are the smallest circulating blood cells. In 1968, Gasic and colleagues discovered a relationship between platelets and tumor metastasis (1). Clinical data suggest that high platelet counts are consistently associated with the increased potential risk of tumor progression in patients with cancer (2). Platelets play an important role in hemostasis and thrombosis; they also promote tumor metastasis through platelet-tumor cell interactions, which are associated with thrombosis formation. Tumor cells induce platelet activation and alter platelet RNA profiles (3). Activated platelets aggregate around circulating tumor cells (CTCs) to form a platelet protective barrier that protects tumor cells from immune destruction. Furthermore, platelets also promote tumor cell migration and invasion of distant organs by inducing tumor cell epithelial-mesenchymal transition (EMT), angiogenesis, anoikis resistance, and extravasation (4). This article reviews platelet-tumor cell interactions and the specific molecular mechanisms. We describe a liquid biopsy technique based on tumor-educated platelets (TEPs). Here, we discuss platelet-based strategies for cancer prevention and tumor-targeted therapy and highlight the opportunities and challenges of aspirin and other platelet inhibitors in cancer therapy.

## 2 Platelet structure and function

Platelets are small, specialized, non-nucleated blood cells. The bone marrow and lung are the sites of considerable platelet production, and the lung is an organ with considerable hematopoietic potential. Functional platelets are 2-5 μm non-nucleated discoid cell components with an average volume of 6-10 femtoliters, and approximately 400 billion circulating platelets are present per liter of blood. The mean survival time of platelets after shedding from megakaryocytes is 5-7 days. The cytoskeleton consists of many cross-linked actin filaments that control the shape of the platelet. The structural rearrangements of the cytoplasm in these cells include forming a dividing membrane system, assembling dense tubular networks, and forming granular platelet components. The platelet surface is covered with a glycoprotein membrane, and the invaginated plasma membrane of platelets increases the effective surface area for absorption of coagulation protein and allows small molecules to pass through and transfer their cytoplasmic contents to other cells. Microparticles and exosomes derived from platelets are biological carriers for intercellular communication (5). Platelets contain approximately 40-80 alpha granules, 4-8 dense granules, and a few lysosomes (6). Alpha granules contain various proteins, including pro-angiogenic and anti-angiogenic factors, whereas dense granules contain small molecular substances such as calcium ions, 5-hydroxytryptamine (HT), and adenosine triphosphate (ATP), and adenosine diphosphate (ADP). Platelet-derived exosomes can deliver specific miRNAs, such as miR-21, miR-223, miR-339, and miR-328 (7), which have potential value in tumor detection and diagnosis (8).

**Figure 1:**
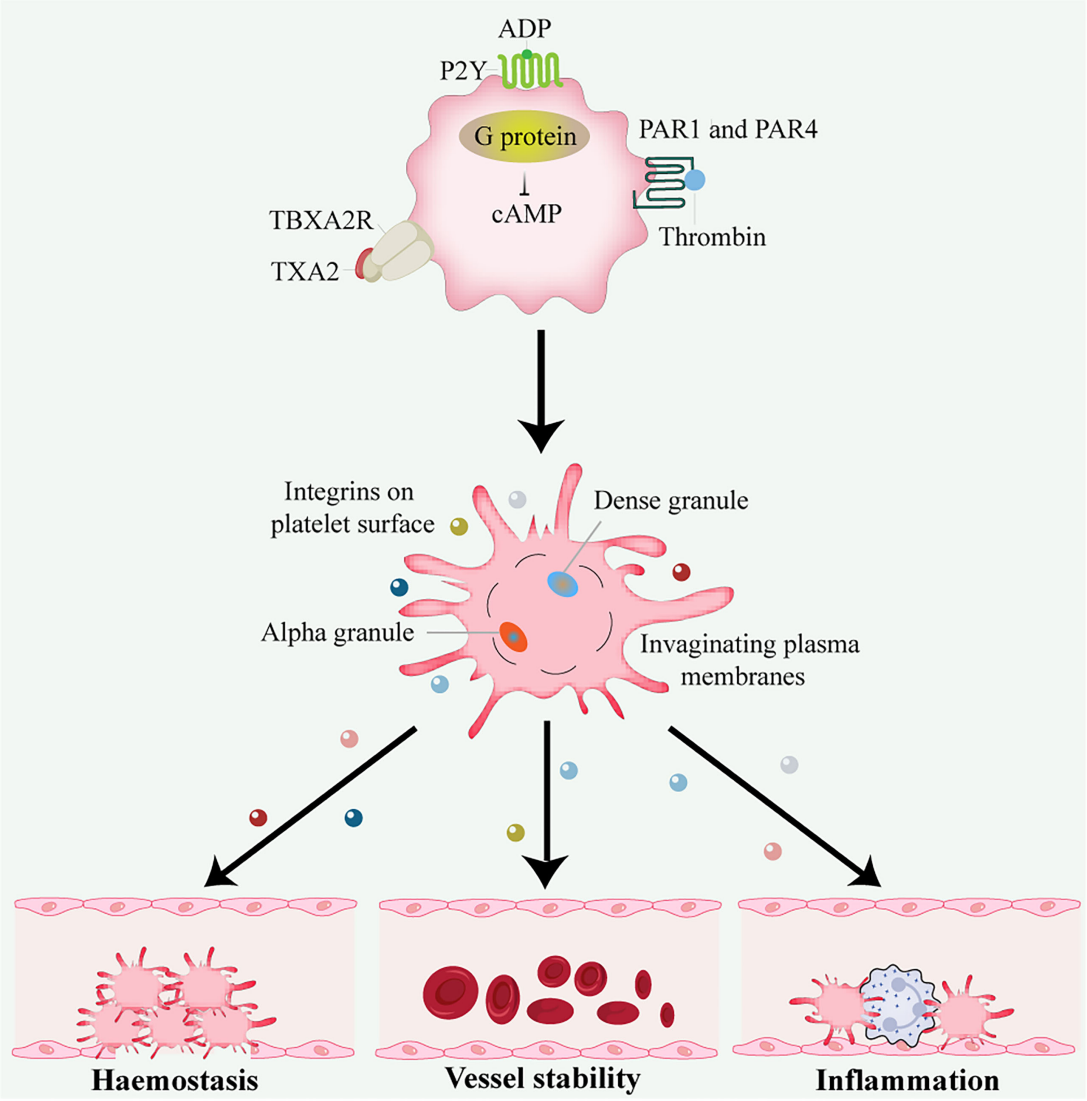
Platelet activation and function. Thrombin activates G protein-associated PARs on the platelet surface during platelet activation, triggering intracellular calcium flux and cAMP reduction. Other platelet activators include subendothelial matrix collagen, ADP, and TXA2 exposed during vascular injury. TXA2 binds to the G protein-coupled receptors P2Y and TBXA2 receptor, stimulating platelet degranulation and release of their contents. Platelets are important in hemostasis and thrombosis, inflammation, wound healing, immune responses, angiogenesis, and vessel stability. ADP, adenosine disphosphate; cAMP, cyclic adenosine monophosphate; PAR, protease-activated receptor; TXA2, thromboxane A2.

In addition to hemostasis and thrombosis, platelets have important roles in many physiological processes, including inflammation, wound healing, angiogenesis, immune responses, cancer, and neurodegenerative diseases (9) (**Figure 1**).

## 3 Role of platelets in cancer dissemination

**Figure 2:**
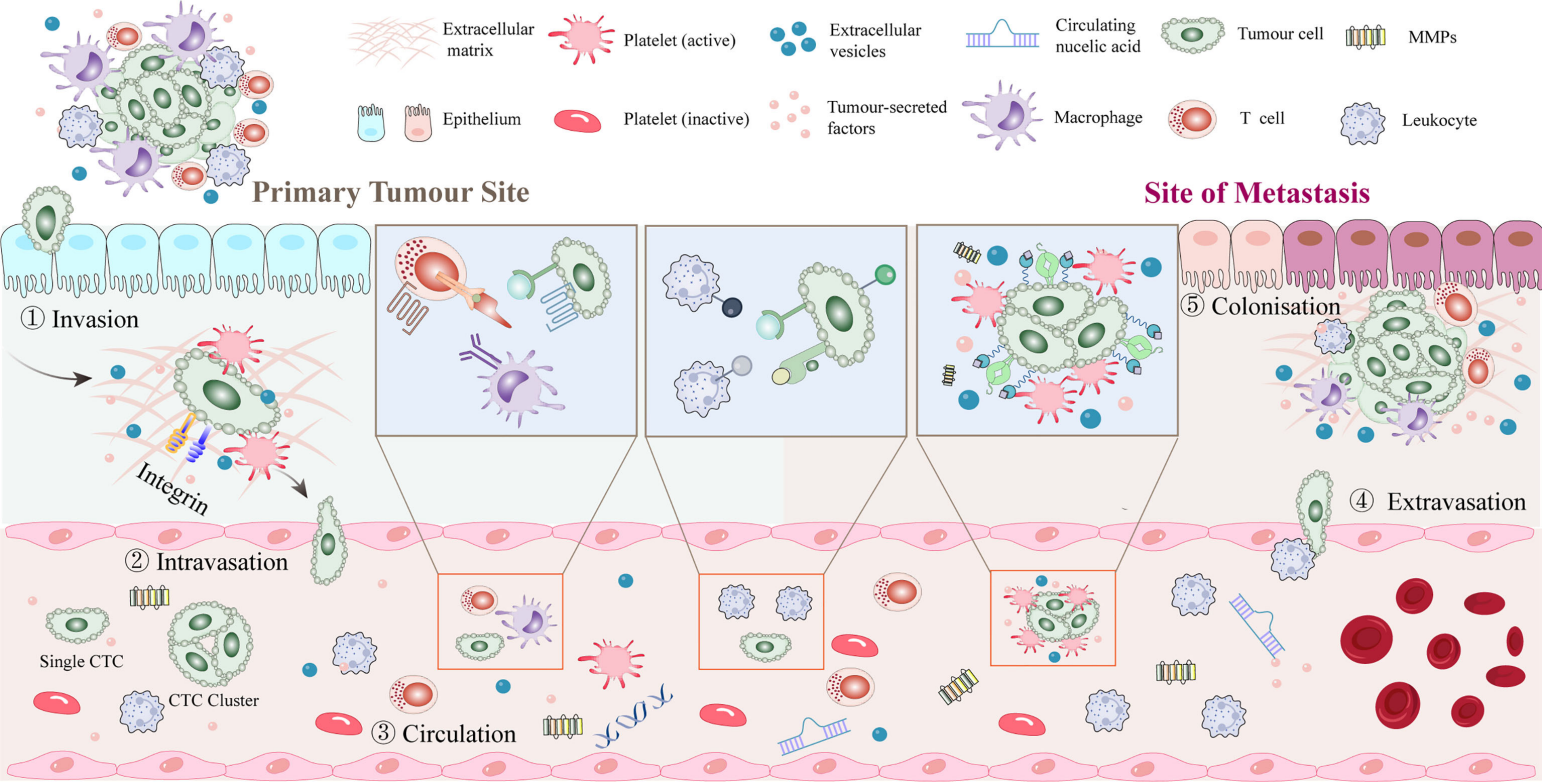
Platelet-tumor cell interactions in early steps of the metastatic cascade. After dissociating from the primary tumor, tumor cells enter the blood vessels and rapidly pass through the circulation into the vasculature of secondary organs. Platelets aggregate around CTCs or around stalled tumor cells to form a platelet protective barrier. Tumor cells promote platelet induction of NETs. Extravasation typically occurs within 1 to 3 days of the initial arrest, seeding into the stroma of the target tissue or organ and recruiting myeloid cells to the early metastatic niche. Tumor cells may remain dormant or start growing again to initiate metastases, with only a few cells completing the metastatic cascade and forming clinically relevant metastatic tumors. CTC, circulating tumor cell; NET, neutrophil extracellular trap.

The metastatic potential of tumor cells is influenced by the tumor microenvironment (TME). Tumor cells first leave their primary growth site. After tumor cells enter the blood circulation system and survive *via* platelet-mediated protection, the CTCs undergo extravasation, anoikis resistance, and angiogenesis. The interaction between tumor cells and platelets plays an important role in all stages of tumor progression (**Figure 2**).

### 3.1 Tumor-cell-induced platelet activation and aggregation

Tumor cell-induced platelet aggregation (TCIPA) is an important process by which tumor cells stimulate platelet aggregation through different mechanisms and release particle contents (10). Almost immediately after CTCs enter the blood circulation, platelets bind to CTCs and form TCIPA around them (i.e., platelet-platelet, platelet-tumor, and tumor-platelet-leukocyte aggregation). The TCIPA protects CTCs from high shear stress and immune surveillance in the bloodstream, promotes tumor metastasis, increases the risk of thrombosis, and is inversely associated with prognosis and survival (11). The ability of different tumor cells to induce TCIPA is related to their different metastatic potential.

**Figure 3:**
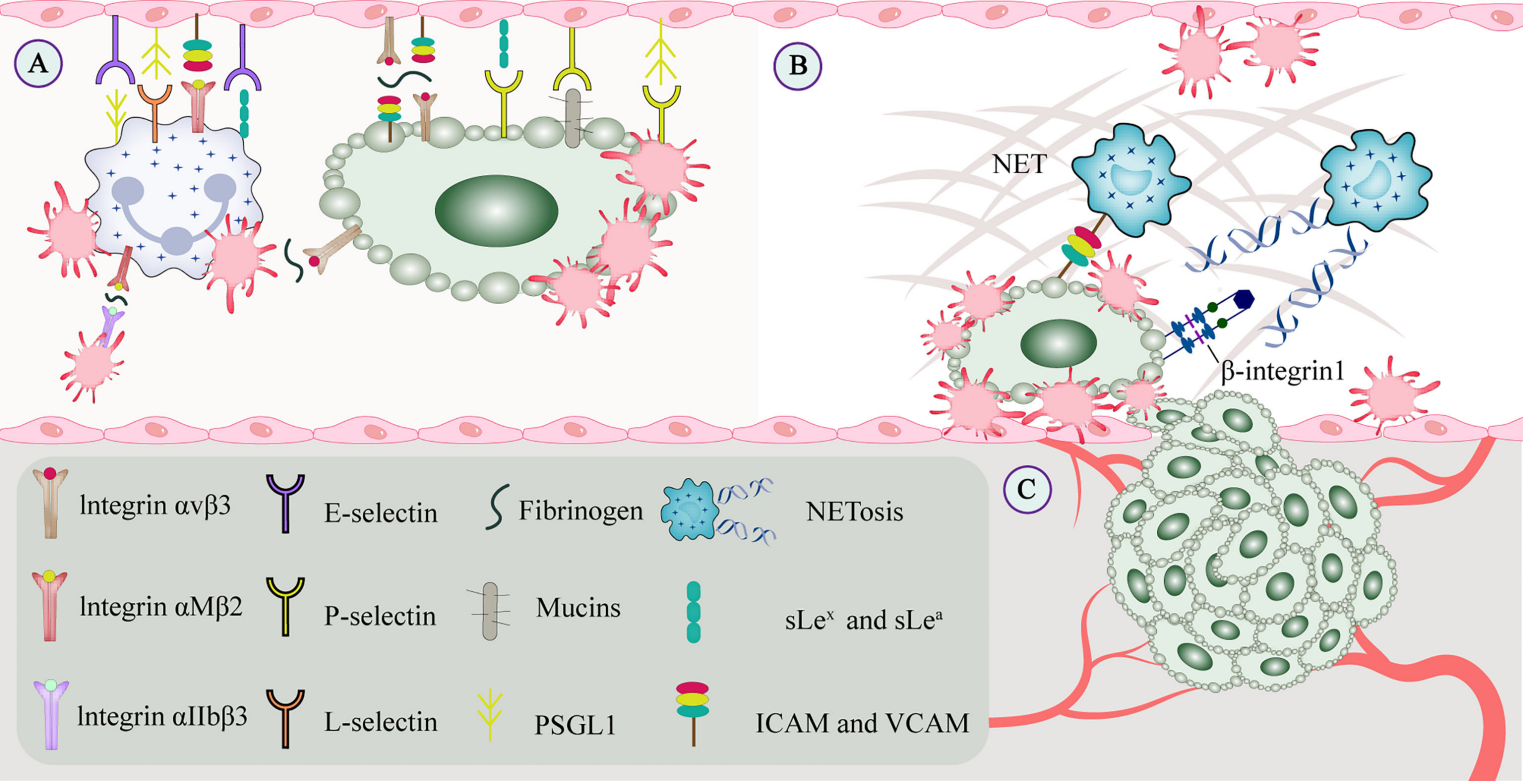
CTCs interact with constituents of the blood circulation. **(A)** Cell adhesion molecules mediate adhesion and signal transduction between cells and between cells and the extracellular matrix to promote tumor metastasis. Integrins and selectins play important roles in this process. On the one hand, selectins can bind to leukocytes or endothelial cells to enable and maintain cell rolling. On the other hand, GPVI and podoplanin can be involved in platelet-mediated tumor metastasis. **(B)** Platelets promote recruit neutrophils to generate extracellular traps, and NETs interact with CTCs to form a protective barrier that facilitates CTC extravasation and the formation of metastatic niches. CTCs promote platelet-induced NETosis, associated with increased complications such as tumor-associated thrombosis, venous thromboembolism, and tumor metastasis. **(C)** Growth factors secreted by platelets (such as VEGF, PDGF, and FGF) bind to the corresponding receptors (such as integrins, Notch signaling receptors), which may regulate tumor angiogenesis and vascular integrity. CTC, circulating tumor cell; EGF, epidermal growth factor; GP, glycoprotein; NET, neutrophil extracellular trap; PDGF, platelet-derived growth factor; VEGF, vascular endothelial growth factor.

TCIPA is associated with a higher risk of thrombosis. On the one hand, in cancer patients, tumor-associated tissue factor expressed by tumor cells interacts with platelet-derived coagulation factors and produces thrombin. Thrombin not only activates platelets and stimulates tumor cell growth, but also promotes the adhesion of CTCs to platelets (12). Thrombin, ADP, and thromboxane A2 (TXA2) interact with G protein-coupled receptors P2Y1 and P2Y12 on the platelet surface and initiate specific downstream signaling cascades (13). Upon activation by thrombin, platelets can release granular factors that promote TCIPA and mediate immune responses (14). On the other hand, C-type lectin-like receptor (CLEC) -2, glycoprotein (GP) VI, and FcRγIIA are tyrosine-activated motif immunoreceptors on platelets. CLEC-2 mediates the binding of its immunoreceptor tyrosine-phosphorylated Hemi-ITAM in the cytoplasm to tyrosine kinase Syk to form a signaling complex (15). GPVI cytoplasmatic tail and the FcRγ cross-link and aggregate *via* a salt bridge, leading to phosphorylation of the ITAM motif in the FcRγ chain and activation of Syk, which downstream assembles LAT and SLP76 signaling complex that ultimately leads to platelet activation and aggregation (16). Tumor microparticles released by tumor cells contain tissue factor and P-selective glycoprotein ligand (PSGL) -1 that bind to platelet P-selectin to activate and recruit platelets (17). Integrin aIIbb3 on the activated platelets binds to the fibrinogen of tumor cells, and outside-in signaling stimulates Rap1b-GTP and phospholipase C and subsequently accelerates platelet activation (18). High-mobility group box 1 released by tumors interacts with toll-like receptor (TLR) 4 on platelets for local platelet activation (19). Platelets are also activated by direct contact with molecules on the tumor cell membrane surface (20) (**Figure 3**).

Tumor growth is accompanied by an increased risk of platelet abnormalities and thromboembolic disease (21). Platelets and coagulation proteins can form protective thrombi around CTCs. A case-control study of 3220 participants showed that the risk of venous thrombosis or embolism was 4–7.5 times higher in patients with malignant tumors versus patients with non-malignant tumors (22). The incidence of venous thrombosis and embolism was higher in patients with pancreatic cancer, brain cancer, gastric cancer (23), or gynecologic malignancies (24). Thrombocytosis has been observed to be associated with poorer progression-free survival and overall survival in lung, colorectal, gastric, breast, kidney, brain, pancreatic, and some gynecologic tumors (2, 25–27).

### 3.2 Platelets promote tumor evasion of immune destruction

Natural killer (NK) cell patrolling is the predominant form of anti-tumor surveillance by the immune system during the cascade of tumor metastasis (28). Cell lysis occurs through different mechanisms: binding of death receptors, secretion of the tumor suppressor interferon (IFN) -γ, and release of cytotoxic particles. Activated platelets can aggregate on the surface of CTCs to form a thrombus and protect the CTC from high shear forces in the blood circulation and from NK cell-dependent tumor cell lysis (29). Tumor cells masquerade themselves as platelets by displaying platelet cell receptors on their surface, which essentially consists of major histocompatibility complex I expressed in high levels by platelets and then transferred to the CTC surface (30). This “platelet mimicry” enables tumors to evade recognition and attack by NK cells (31). NK group 2 member D (NKG2D) is the only *in vivo* receptor that can recognize soluble major histocompatibility complex I molecule-associated protein A/B, which can inhibit NK cell toxicity and anti-tumor activity (32). TEPs secrete large amounts of platelet-derived growth factors (PDGFs), such as IFN-γ or transforming growth factor (TGF) -β. TGF-β downregulates the expression of the NKG2D receptor and thereby reduces the anti-tumor effect of NK cells (33). Platelet-derived ADAM10 can regulate NKG2D receptor expression by NK cells and promote NKG2D ligand release (34). Similarly, TGF-β is closely related to the overexpression of glucocorticoid-induced TNF receptor-related ligand in activated platelets that can interact with related receptors on NK cells and inhibit their action (35). TGF-β also inhibits NK cell function by inhibiting the mammalian target of rapamycin activity (36).

**Figure 4:**
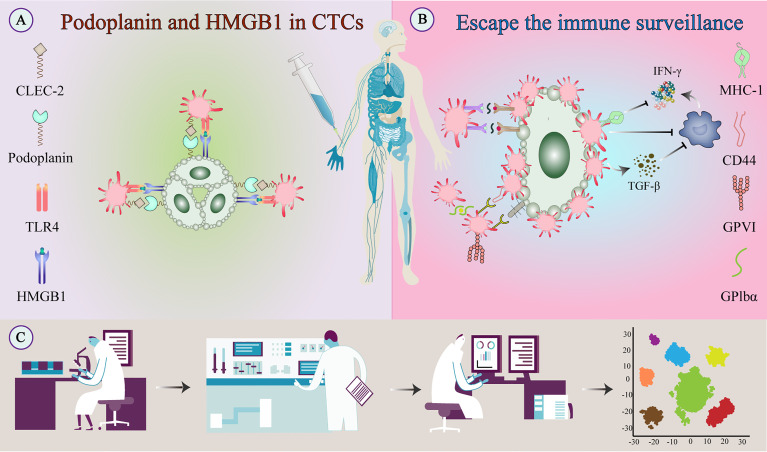
Escape of tumor cells from immune surveillance. **(A)** Platelets bind to tumor-derived HMGB1 *via* TLR4 and interact with podoplanin-expressing tumor cells *via* CLEC2 to stimulate their activation. **(B)** Activated platelets and fibrinogen can form a protective barrier against the mechanical forces of blood flow and also against NK cell attack. In addition to providing physical shielding, platelets can protect CTCs from circulating NK cell-mediated tumor lytic activity and can interfere with tumor cell recognition by NK cells. **(C)** TEPs as a noninvasive biomarker source for cancer detection and progression monitoring. The examination of TEPs mainly includes ultra-deep, massively parallel, and long-read sequencing of TEP transcripts and possible detection of epigenetic transcriptional signatures. Improving the sensitivity and accuracy of detection techniques and the specific selection of tumor-affected platelets can improve diagnostic accuracy and treatment prediction. CLEC-2, C-type lectin-like receptor-2; CTC, circulating tumor cell; HMGB1, high-mobility group box 1; NK cell, natural killer cell; TEPs, Tumor-educated platelets; TLR, toll-like receptor;.

In addition to NK cells, platelet-derived TGF-β converts CD4+ T cells into inducible regulatory T cells and exerts anti-tumor immunity by attenuating tumor-infiltrating lymphocytes (37). In head and neck squamous cell carcinoma, platelets can reduce the expression of programmed cell death protein 1 (PD-1) on CD4+ T cells and inhibit the production of IFN‐γ and TNF-α cytokines (38). Platelets may also inhibit IFN-γ production and T cell proliferation by releasing lactate (39). Platelets reduce tumor cell recognition and cytotoxicity by NK cells by reducing the expression of CD112 and CD155 on tumor cells and their related receptors CD226 and CD96 on the NK cells (40) (**Figure 4**).

### 3.3 Platelet interactions with neutrophils of the tumor microenvironment

Platelets and neutrophils are two key players in the early innate immune response in blood circulation. Neutrophils in the TME expel intracellular contents and DNA in cobweb-like chromatin structures in the form of neutrophil extracellular traps (NETs). Cell death occurring by NADPH oxidase mediated by NET capture is known as NETosis (41). NETs have been identified as an important factor in tumor-associated thrombosis, venous thromboembolism, and tumor metastasis (42). Neutrophils can cause histone citrullination by the enzyme peptide arginine deiminase 4 (43). NET activates platelets by releasing norepinephrine (NE), citrullinated histone H3 (H3Cit), and myeloperoxidase (MPO), all three of which are the most reliable markers of persistent NETosis (44). High levels of H3Cit have been observed in the plasma of cancer patients and have been associated with poor prognosis (45).

Extracellular DNA in NETs affects platelet activation and its prothrombotic function by enhancing the TCIPA formation (46), and tumor-activated platelets are a pro-NETosis component in the TME (47). Similar to the platelet protective barrier, NETs interact with CTCs *via* β1-integrin to form a protective barrier, thereby promoting the survival and extravasation of CTCs in the blood circulation (48). Cytokines expressed by tumor cells, such as granulocyte colony-stimulating factor and interleukin (IL)-8, directly induce NETosis in tumor cells (49). Similarly, tumor-derived fibronectin ED-A stimulates platelet aggregation and thrombosis through TLR4 and promotes platelet-induced NETosis (50). Furthermore, lipopolysaccharide stimulation of platelets or tumor-derived factors *via* TLR4 enhances platelet-neutrophil adhesion and induces NETosis, but does not promote platelet aggregation or upregulate P-selectin expression (51). Activated platelets expose P-selectin on the surface, which interacts with the neutrophil PSGL-1 receptor, promoting neutrophil activation and thrombosis (52). Studies have shown that in mice overexpressing P-selectin, platelets can induce NETosis, whereas, in those deficient, tumor angiogenesis cannot be induced (53) (**Figure 3**).

### 3.4 Platelets induce an invasive epithelial-mesenchymal transition phenotype of tumor cells

The metastatic potential of tumor cells is closely related to EMT; tumor cells gradually lose epithelial identity and morphology and acquire mesenchymal properties such as cell motility, invasiveness, and resistance to apoptosis (54). Platelet-treated tumor cells show downregulated E-cadherin levels (55) and upregulated expression of mesenchymal markers such as Snail, vimentin, fibronectin, plasminogen activator inhibitor 1, and matrix metalloproteinase (MMP)-9. Transferred signaling is thereby activated in platelet-treated tumor cells (56).

Tumor cell invasion-metastasis cascade is initiated *via* the TGF-β/Smad and NF-κB signaling pathways activated by platelet-derived TGF-β. The TGF-β/Smad pathway can also be activated by direct contact with integrin α2β1 (37). CLEC-2 on platelets binding with podoplanin on tumor cells can induce platelet release of TGF-β and promote EMT (57). Inhibition of podoplanin inhibits metastatic progression in lung squamous cell carcinoma (58). Platelet-mediated EMT of tumor cells is promoted by many microRNAs and other mediators released by platelets in addition to TGF-β, including prostaglandin E2 (PGE2), PDGF, and lysophosphatidic acid (LPA) (59). PGE2 promotes tumor cell EMT and invasion by interacting with oncogenic signals, including epidermal growth factor (EGF) and epidermal growth factor receptor (EGFR) (60). In renal cancer, PGE2 promotes progression by activating the cyclic AMP (cAMP)/protein kinase A (PKA)-cyclooxygenase2 (COX2) signaling pathway (61). Thrombospondin (TSP) 1 and platelet-derived PDGF promote tumor invasion and metastasis by upregulating MMP2/MMP9 expression and inducing EMT through the p38 MAPK signaling pathway (62, 63). Platelet-derived LPA stimulates the secretion of IL-6 and IL-8 by activating the autotaxin (ATX)/LPA signaling axis, which suppresses immune responses and promotes cancer cell invasion and metastasis, upregulates oxidative stress and drug resistance-related gene expression, and stabilizes nuclear factor-like 2 to induce chemoresistance (64). Platelet-derived LPA, in turn, is promoted by the interaction of platelets with tumor cell CD97 and subsequent activation of platelets through LPA-mediated signaling (65). Both tumor mesenchymal stem cells and tumor-activated platelets may release the chemokine ligand 5 (CCL5) (66). CCL5 and EGF can induce tumor cell invasion by promoting IL-8 secretion from tumor cells *via* Akt signaling activation (67). CCL5 released by tumor-activated platelets mediates migration and chemotaxis of T lymphocytes and monocytes, and anti-chemokine receptor-5 therapy causes tumor-associated macrophages to switch from pro-tumor to anti-tumor roles in patients with liver metastases (68).

EMT plays an important role in tumor resistance, recurrence, and metastasis. EMT is also said to contribute to treatment resistance because of reversible epigenetic changes observed in acquired chemoresistance due to molecular and phenotypic links that exist between drug resistance and EMT changes in tumor cells. EMT may also promote radio-resistance in multiple tumors, potentially providing important new avenues for the development of new therapeutic strategies (69, 70).

### 3.5 Platelets facilitate tumor anoikis resistance and extravasation

Extravasation usually occurs within 1-3 days of tumor cells entering the circulation. Anoikis is programmed cell death resulting from the detachment of cells from the extracellular matrix (ECM) (71). Before metastasis, CTCs lose their platelet protective barrier during extravasation. Successful metastasis of tumor cells requires the formation of an early metastatic niche to acquire an anoikis resistance (72).

Platelet-derived PDGF, LPA, and 12-hydroxyeicosatetraenoic acid (12-HETE) promote the release of MMPs from the CTCs (73). During metastasis, MMPs and platelets decompose most of the ECM components and increase endothelial cell permeability, and the exposed collagen proteins induce platelet recruitment to endothelial cells (74). Additionally, ATP binds to the vascular endothelial receptor P2Y2 and increases endothelial permeability (75). Furthermore, platelet-derived ATX can convert lysophosphatidylcholine to LPA (76). which then binds to LPA receptor-1 on the CTC, enabling tumor cells to acquire anoikis resistance by induction of the RhoA-Gα12/13-YAP-1 signaling (77). Additionally, PDGF-BB can promote pancreatic tumor cell anoikis resistance through YAP signaling (78).

Platelets release chemokines or cytokines, including TGF-β, platelet factor 4/C-X-C motif ligand (CXCL) 4, CXCL7, stromal cell-derived CXCL12, vascular endothelial growth factor (VEGF), and CCL5, to promote the activation of endothelial cells or induce the recruitment of bone marrow-derived cells to distant organs (79). CCL and CXCL promote vascular extravasation by recruiting lymphocytes, granulocytes, and monocytes to form an early metastatic niche (80). TGF-β, expressed by tumor cells or platelets, initiates the early metastatic niche and growth of fibroblasts at metastatic sites by enhancing the expression of the ECM proteins periostin and tenascin (81). Similarly, VEGF-A released by CTCs increases the permeability of vascular endothelial cells *via* von Willebrand factor secretion (82).

### 3.6 Impact of platelets on tumor angiogenesis and vascular remodeling

When tumors reach a critical size of a few cubic millimeters, they induce angiogenesis for the supply of oxygen and nutrients and the removal of metabolic waste. Most of the angiogenesis-related factors in the circulation are released by platelets, thereby regulating tumor angiogenesis and maintaining vascular integrity (83). Platelets can actively synthesize proteins upon stimulation, or megakaryocytes can selectively transfer a subset of proteins or mRNAs to platelets (84). Platelets take up these factors by endocytosis and segregate them into distinct α-granules, such as VEGF, PDGF, basic fibroblast growth factor, and EGF (85). VEGF is one of the most important proteins stimulating angiogenesis in distant metastases (86).. Platelets activate selective protease-activated receptor (PAR) 1 to stimulate the release of VEGF-containing particles while reducing angiogenic endostatin expression. Conversely, platelets activate selective PAR4 to stimulate the release of endostatin-containing particles (87).

Thrombin is a key factor involved in tumor angiogenesis. Thrombin induces the proliferation of transformed cells and enhances VEGF expression by activating the PAR-1 receptor (88). ADP stimulation of platelets also promotes VEGF release and inhibits endostatin release, thereby favoring tumor angiogenesis (89). The CD40 on platelets also promotes angiogenesis (90). Furthermore, platelet-secreted CXCL12 can recruit hematopoietic progenitor cells and thus promote angiogenesis (91). Along with regulating tumor angiogenesis, platelets also regulate tumor vascular integrity by inhibiting immune cell invasion into the tumor tissue. Platelet-secreted 5-HT maintains tumor vascular stability by counteracting tumor cell-derived VEGF (92) (**Figure 3**).

## 4 Tumor-educated platelets as biomarkers of cancer

### 4.1 Tumor-educated platelets transcriptomes

Tumor cells can induce alterations in the platelet transcriptome profile either directly (by transferring tumor-derived RNA) or indirectly (by releasing signals that regulate platelet mRNA processing), thereby stimulating protein synthesis to generate tumorigenic platelets (93)​​. Platelets can uptake circulating proteins and several types of RNA, including mRNA, miRNA, circRNA, lncRNA, and mitochondrial RNA, from the TME (94). Platelet microparticles serve as intercellular carriers to deliver mRNA regulatory Ago2•microRNA complexes to endothelial cells and potentially to other recipient cells in the circulation (95). This communication model suggests that the platelet transcriptome can influence gene expression in recipient cells (93). Tumor cells “educate” platelets in at least the following three ways: 1) by inducing protein translation and subsequent RNA decay; 2) by stimulating specific RNA splicing events; 3) by isolating and releasing RNA from the circulation (56). TEPs are advantageous as biomarkers because they are abundant, are easy to isolate, and possess the ability to process RNA in response to external signals (96). Other platelet-derived liquid biopsy biomarkers include platelet count, artificially educated platelets, and platelet RNA and proteomic analysis.

### 4.2 Emerging technologies for tumor-educated platelets biomarkers

Platelets are a source of RNA biomarkers that exchange information. Detection of tumor-derived RNA requires ultra-deep massive parallel sequencing or targeted methods, droplet digital polymerase chain reaction (PCR), real-time PCR, or amplification refractory mutation system PCR. Therefore, an optimal biomarker-to-background ratio is required to ensure the identification and development of tumor biomarker assays. The transcriptome potential of diagnostic TEPs was first investigated in 2010 by using microarray analysis on platelet mRNA from metastatic lung cancer patients and healthy donors, which found that platelets can induce tumor growth and progression by releasing epigenetic silencing factors (97). Nilsson et al. found tumor-derived PCA3 transcripts and EGFRvIII in platelets isolated from prostate cancer and glioma patients, respectively, which demonstrated that tumor cells could transfer RNA into platelets (98). Davizon et al. demonstrated the remarkable stability of platelet RNA over time (99). Altered spliceosome function in cancer may explain the distinct splicing events observed in the TEP RNA signature profile (100). The efficiency of surrogate TEP RNA signature in detecting mutations was evaluated by comparing targeted deep amplification sequencing of KRAS and EGFR mutations. The wild-type/mutation ratio of specific mutant tumor markers detected in plasma was found to be more reliable (101)​​. Importantly, TEP mRNA sequencing was able to identify cancer with 96% accuracy and distinguish six primary tumor types with 71% accuracy, including non-small cell lung cancer, glioblastoma, colorectal cancer, pancreatic cancer, hepatobiliary cancer, and breast cancer (101).

This RNA modification in the TEP transcriptome may represent a new diagnostic biomarker. Another potential biomarker found in platelets is circRNA, produced from precursor mRNAs by a back-splicing mechanism. CircRNAs can act as sponges for miRNAs and RNA-binding proteins. CircRNAs can be selectively released into vesicles by platelets and may be involved in signaling pathways (102). Compared to several types of nucleated cells, circRNAs are 17-188-fold enriched in platelets (103). Furthermore, open reading frames in circRNAs can be translated to produce functional polypeptides (104). Third-generation or long-read sequencing may be another potential approach to improve the assessment of variants present in platelet transcriptomic profiles. Oxford Nanopore Technologies has now developed a direct sequencing protocol for RNA molecules, and this protocol is immune to reverse transcription and RNA amplification and enables the detection of epitranscriptomic base modifications (105).

As mentioned earlier, combining liquid biopsy sources for cancer detection and localization is effective. In patients with metastatic colorectal cancer and upper urinary tract cancer with a tumor diameter >3 cm, the platelet/lymphocyte ratio was found to be correlated with tumor location and prognosis (106, 107). In patients with oral and lingual squamous cell carcinoma, the platelet-to-neutrophil ratio can predict lymph node metastasis (108). Further research is needed to understand the mechanism of RNA transfer between platelets and tumor cells; this would include the use of mouse models, megakaryocyte analysis, and platelet RNA and proteomic multi-omics analysis. Tumor cells are injected intravenously into experimental mice, which is generally considered a standard model for studying hematogenous spread. Despite some limitations (for example, no primary tumor, tumor cells are injected in a single event rather than dispersed over a long period, etc.), this experimental setup also provides fundamental advantages: 1) it allows close temporal monitoring of early interactions between individual tumor cells and the host TME; 2) it allows precise characterization of specific steps of the metastatic cascade affected by specific experimental treatments (109). Zaslavsky et al. demonstrated that tumor education of macrophages could affect platelet content (110). Bone marrow macrophages show high expression of anti-angiogenic TSP1, which may be responsible for the different TEP phenotypes (111). An important method to study the platelet release *in vitro* is mass spectrometry-based proteomics, including targeted analysis, post-translational modification, and multi-omics approaches (112). In ovarian cancer, the multi-omics analysis identifies the key genes associated with N6-methyladenosine RNA modification and are mainly involved in the platelet activation pathway (113). Designing extensive prospective studies to statistically validate individual TEP markers or their RNA signatures can help us better understand and monitor cancer progression (**Figure 4**).

## 5 Therapeutic use of platelet inhibitors and targeted drug delivery platform

Platelet-based tumor-targeted therapy strategy consists of a targeted drug delivery platform for tumor therapy by developing antiplatelet drugs and by using platelet-derived “nano-platelets”. Many drugs targeting platelet receptors, interfering with the release of platelet particles, or inhibiting platelet-specific enzymes are already in clinical use or preclinical development (**Table 1**).

**Table 1:** Platelet inhibitors and anticoagulants in cancer therapy.

Receptor	Ligand	Pathway	Drug
GPIb(GPIX,GPIbβ,GPIbα,GPVcomplex)	VWF	FAK/Factin,CaM/Lyn/Syk/SLP76/Btk/PI3K/PLCγ2	GSK2256098^C^, PF562271^PC^, Y15 & Y11^PC^, CEP37440^PC^ defactinib^C^, PF573228^PC^, H6B4^PC^, NIT family mAb^PC^
GPIIb/IIIa (αIIb/β3)	fibrinogen	FAK/CIB-1/actin, ARP 2/3/actin polymerization,FAK/paxillin/RhoGEF	abciximab^C^, eptifibatide^C^, tirofiban^C^, mAb10E5^PC^, XV454^PC^
GPVI	collagen	CaM/Lyn/Syk/SLP76/Btk/PI3K/PLCγ2	losartan^PC^, scFv 9012^PC^, revacept^C^
CLEC-2	podoplanin	Syk/PLCγ2	2CP^PC^, Mabs
P-selectin	mucins, P-, E-, L-selectins	Shc·Grb2·Sos1	rivipansel^C^, Anti–P-selectin antibody^PC^, crizanlizumab^C^, anti-CD24 antibody FL80^PC^, heparins^C^
αvβ3	fibrinogen vitronectin	c-Src, FAK, paxillin, PI3K	SB-273005^PC^, SC-68448^C^, EMD121974^C^, vitaxin^C^
PAR1 antagonist	ADP	GPCR/G_q_/RhoGEF/Rho/ROCK/LIMK/cofilin/actin/MLCK/myosin,β-arrestin	voraxopar^C^, CH79797^C^, RWJ 56110^PC^
P2Y_12_ Receptor antagonists	ADP	Ga_i2_/AC	clopidogrel^C^, ticlopidine^C^, prasugrel^C^, ticagrelor^C^, cangrelor^C^, elinogrel^C^
EP3, prostaglandin E_2_ receptor antagonist	PGE_2_	GPCR/G_αq_/PLCγ/P/IP_2_/IP_3_/IP_3_R/Ca^2+^release/DAG/PKC/CalDAGGEF1/Rap1B/RIAM/actin	DG 041^*PC*^

*^PC^, pre-clinical; ^C^, clinical.*

### 5.1 Platelet inhibitors and anticoagulants in cancer therapy

#### 5.1.1 Aspirin use in cancer therapy

Aspirin inhibits COX isoform-related inflammation and apoptosis and inhibits the release of MMPs from platelets, thereby preventing the degradation of ECM and reducing CTC invasion (114). Additionally, aspirin may also inhibit the IκB kinase β signaling and extracellular signal-regulated kinase (115). Aspirin inhibition depends on the dose, duration, and even cancer type.

In 2007, the United States Preventive Services Task Force recommended against the routine use of aspirin to prevent any cancers (116). The ARRIVE trial (Aspirin Reduces Risk of Initial Vascular Events) found that routine use of aspirin increases cancer incidence (117). Results of the ASPREE trial (Aspirin in Reducing Events in the Elderly) showed that there were 127 lower GI bleeds (73 in aspirin and 54 in the placebo arm, HR 1.36, 95% CI 0.96 to 1.94, p=0.08) and 137 upper GI bleeds (89 in aspirin arm and 48 in the placebo arm, HR 1.87, 95% CI 1.32 to 2.66, p<0.01). Long-term use of low-dose aspirin in healthy older adults substantially increased the risk of gastrointestinal bleeding (118). However, these trials had certain limitations: For example, as follow-up was < 5 years, the benefit of aspirin in reducing tumorigenesis may not have emerged; also, the study subjects in both trials were older than 70 years and had a higher incidence of early-stage undiagnosed cancers. ASPREE-XT (Aspirin in Reducing Events in the Elderly Extension observational cohort study) is an ongoing follow-up observational study of the ASPREE trial to study the effect of aspirin on subjects of different ages and tumor stages (118). Two ongoing trials are expected to be completed in 2026: the ASCOLT (Anglo-Scandinavian Cardiac Outcomes Trial) investigating the effect of aspirin on post-surgical prognosis and standard chemotherapy in Dukes B and C colorectal cancer patients, and the Add-Aspirin trial investigating disease recurrence and survival after primary therapy for non-metastatic solid tumors (119).

#### 5.1.2 Platelet P-selectin

Selectin is a transmembrane cell adhesion molecule expressed by platelets, endothelial cells, and leukocytes. Selectins recognize ligands containing the tetrasaccharide sialic acid Lewis a and x antigens, which are upregulated in many tumors. P-selectin present in platelets has two major ligands, PSGL-1 and CD44 (120). Following platelet activation, P-selectin is released from alpha granules and translocated to the platelet membrane (PM), where it is activated upon ligand binding.

P-selectin plays a key role in tumor proliferation, angiogenesis, and EMT by acting as a platelet molecular switch to promote platelet adhesion and aggregation (121). Furthermore, P-selectin promotes tumor metastasis by functional inhibition of CD4+ and CD8+ T cells *via* infiltration of regulatory T cells and aggregation of other cells (122). The binding of P-selectin to leukocytes can support and maintain cell rolling. Non-mucin ligands of P-selectin regulate platelet binding to neutrophils to form NET, and NET can recruit platelets and aid in immune escape and extravasation of CTCs (123).

In mouse models, inhibiting P-selectin can reduce tumor metastasis (124). P-selectin knockdown in mice with colon adenocarcinoma resulted in reduced platelet-tumor cell aggregation and, therefore, metastasis (125). Platelet P-selectin inhibitors have been used clinically (126), but it remains to be assessed whether they are safe, effective, and tolerable in cancer.

#### 5.1.3 Platelet integrins

Integrins are heterodimers composed of alpha and beta subunits (127). The four integrins identified in TEPs to play roles in tumor progression include αIIbβ3, αvβ3, α6β1, and α2β1 (128). Integrin αvβ3 binds to fibrinogen and promotes the formation of cross-linked barriers by platelets on the surface of CTCs (129). Integrin αvβ3 binds to tumor cell CD97 and promotes ATX-mediated anoikis resistance (130). Furthermore, integrin α6β1-dependent platelet-tumor cell interaction promotes the release of platelet granules and increases the expression of MMP-1 and MMP-2 in CTCs, favoring the extravasation of tumor cells (131). The interaction between integrin α6β1 and MMP-9 expressed by CTCs promotes efficient lung cancer metastasis (132).

Integrin αIIbβ3 plays a central role in platelet activation, adhesion, aggregation, and thrombus consolidation (133). Activation of integrin αIIbβ3 is dependent on the stimulation and regulation of talin and kindlin cytoplasmic proteins (134). such as GPVI, GPIB-IX-V, TXA2, thrombin, and ADP (135). GPVI forms a cross-linked chain with integrin αIIbβ3 on the CTC surface binds to the collagen to form a cross-linking barrier, and binds to Galectin-3 expressed by CTCs to promote the secretion of TGF-β, EGF, and PDGF (136). Antiplatelet GPVI antibodies cause tumor bleeding without systemic bleeding complications (4). JAQ1F(ab′)2 treatment can inhibit the expression of GPVI and thereby significantly inhibit the platelet cross-linking barrier formation and tumor metastasis (75).

In a mouse model, platelet integrin αIIbβ3 antagonists inhibited hematogenous metastasis to the same extent as after platelet depletion. However, long-term use of integrin αIIbβ3 inhibitors increases the risk of bleeding (137). Currently, molecular imaging of the active conformation of integrin αIIbβ3 on platelets has been used to detect activated platelets noninvasively. Clinical safety and efficacy of bivalent humanized nanobodies and aptamers with high binding affinity for von Willebrand factor to block platelet aggregation is being evaluated (138). Receipt (AdvanceCOR) is a soluble fusion protein that, at clinically relevant concentrations, effectively prevents platelet-induced upregulation of COX-2 and EMT markers in cancer cells without reducing the expression of platelet GPVI receptors or affecting platelet counts (139). Abciximab is a chimeric human/mouse antibody directed against the integrin αIIbβ3 receptor that binds to integrin αvβ3 on the tumor and endothelial cells leading to integrin αMβ2 expression on leukocytes. This cross-reactivity on both platelets and endothelial cells may contribute to the inhibition of tumor angiogenesis (140).

#### 5.1.4 Platelet CLEC-2

Podoplanin is a mucin-type transmembrane protein, also known as D2-40, M2A, and AGGRUS (141). Podoplanin on tumor cells is the only known endogenous ligand of CLEC-2, a platelet receptor. CLEC-2-expressing platelets can be aggregated and activated through the Src and PLCγ-2 signaling pathways (142). Podoplanin is widely expressed in the brain, lung, heart, kidney, bone, and lymphoid organs (143), and high expression of podoplanin is associated with an increased risk of venous thromboembolism in tumor patients (144). Podoplanin is widely used as a specific marker of lymphoid organs and lymphatic vessels in the TME, and the number of Podoplanin-positive vessels in the TME is often used as a diagnostic marker (143).

In the TME, podoplanin promotes platelet degranulation and regulates signal transduction. Podoplanin-expressing tumor cells have promoted hematogenous metastasis through the CLEC-2-induced platelet aggregation (145). Podoplanin is upregulated in cancer-associated fibroblasts (CAFs) and immune cells of tumor stroma in adenocarcinoma and colorectal cancer (146). Although associated with poor patient outcomes (147), the effect of podoplanin expression in CAFs may depend on the type of tumor cells and the tissue from which the CAF originated. One study found that podoplanin expression in colonic CAFs had a better prognosis and podoplanin knockdown in transwell assays enhanced tumor cell invasiveness (147). *In vitro* studies demonstrated that forced expression of podoplanin in podoplanin-deficient cells resulted in a more mesenchymal phenotype and promoted tumorigenesis and metastasis (143).

CLEC-2 inhibitors can potentially serve as new anti-tumor therapeutic agents. For example, injecting podoplanin-positive melanoma cells into the tail vein of mice pretreated with CLEC-2 antibody resulted in a significant reduction in CTCs, lung tumor niches, and intratumoral thrombus (146). In addition, a podoplanin-competitive platelet antagonist also showed anti-metastatic properties (148). Although blocking podoplanin inhibits tumor cell metastasis, platelet-targeting CLEC-2 inhibitors might be preferred as they reduce hematogenous metastasis rates without a significant increase in bleeding risk (149) (**Figure 4**).

#### 5.1.5 Platelet PAR1 inhibitors

PAR1 is a GP-coupled transmembrane protein highly expressed on platelets (150). In many tumors, PAR-1 expression levels correlate with poor prognosis (151). PAR-1 inhibitors can inhibit tumor metastasis by reducing the number of platelets and targeting tumor cells (152). Some small molecule inhibitors of PAR1 include Vorapaxar, Atopaxar, and PZ-128; PZ-128 may be associated with a reduced risk of bleeding (153). In a breast cancer model, silencing of PAR1 expression with short interfering RNA or treatment with pepducin PZ-128 inhibited PAR1-GP signaling and, therefore, PAR1-related lung (154). However, PAR-1 inhibitors showed opposite effects in pancreatic cancer and could not limit tumor development (155).

#### 5.1.6 Platelet P2Y12 inhibitors

The platelet surface GP-coupled receptor P2Y12 interacts with tumor-derived ADP and initiates specific downstream signaling cascades to induce platelet activation (13). Among P2Y12 receptor antagonists, clopidogrel is the most widely used drug for the treatment of vascular disease, whereas prasugrel and ticagrelor are more potent and have a faster onset of action (156). In an orthotopic mouse model of pancreatic cancer, clopidogrel at a dose of 8 mg/kg completely inhibited ADP-induced platelet aggregation, thus inhibiting cancer-related thrombosis and tumor development and metastasis (157). In a mouse model of ovarian cancer, transgenic knockout of *P2Y12* in platelets or treatment with ticagrelor resulted in a >60% reduction in the growth of orthotopic (158). Despite these beneficial effects of P2Y12 inhibition in a mouse cancer model, another trial showed no difference in cancer rates between the clopidogrel and prasugrel treatments (159). P2Y12 receptor antagonists and PAR1 inhibitors may increase cancer risk, possibly because of impairment of platelet natural barrier function, which can lead to increased vascular permeability and extravasation of tumor cells through circulation or metastatic sites (160). Long-term treatment with clopidogrel and ticagrelor also showed a significant increase in cancer-related deaths in the DAPT (dual antiplatelet therapy) and PEGASUS-TIMI 54 (Prevention of Cardiovascular Events in Patients with Prior Heart Attack Using Ticagrelor Compared to Placebo on a Background of Aspirin-Thrombolysis in Myocardial Infarction 54) trials (161).

### 5.2 Platelet-based targeted drug delivery platform

Platelet-mediated drug delivery systems consist of nanoparticles that target platelet cell adhesion molecules to bind to platelets; nanoparticles delivering antithrombotic or anti-cancer drugs in this manner can act as powerful targeted therapies (162). Major modalities include PM coating, platelet engineering, synthetic platelets, and platelet-triggered drug release (163).

Synthetic silica particles with PM coating specifically induce tumor cell apoptosis through TNF-related apoptosis-inducing ligand (TRAIL) on the surface (164). In addition, a PM-coated core-shell nanocarrier (TRAIL-Dox-PM-NV) carrying TRAIL and doxorubicin can aggregate on the surface of CTCs and inhibit their survival and metastasis *via* P-selectin interaction with CD44 (165). PM-coated core-shell nanocarriers carrying doxorubicin and indocyanine green effectively inhibited growth and metastasis of breast cancers with adequate blood supply but were ineffective in ischemic pancreatic cancer (166). PM-camouflaged magnetic nanoparticles trigger ferroptosis, which induces tumor-specific immune responses and also effectively repolarize macrophages from an immunosuppressive M2 to an anti-tumor M1 phenotype, crucial in metastatic tumors. Sustained tumor elimination was achieved in a murine model (167).

Platelet engineering includes processes such as platelet phagocytosis, platelet surface modification, and drug loading through genetic manipulation (163). The TME promotes platelet activation and release of platelet-derived microparticles. Platelets conjugated to programmed death-ligand 1 (PD-L1), a checkpoint inhibitor, by implanting hyaluronic acid hydrogels, when released into the tumor lumen of mice with resected subcutaneous melanoma tumors successfully inhibited local tumor recurrence as well as distant tumor growth (168). Platelet PD-L1 can also inhibit CD4+ and CD8+ T cells. The data suggest that platelet PD-L1, reflective of the collective tumor PD-L1 expression, plays an important role in the immune evasion of tumor cells. This method of platelet engineering overcomes the limitations of histological quantification of PD-L1 expression within the often-heterogeneous tumors and can help predict the immunotherapy response in non-small cell lung cancer (169). Recombinant platelets expressing PD-1 constructed through platelet engineering technology can effectively aggregate at surgical resection sites and inhibit tumor recurrence *via* PD-1/PD-L1 interaction and thrombus formation. Cyclophosphamide carried by recombinant platelets promotes the anti-cancer effects of CD8+ T cells (170, 171). Designed using genetic manipulation technology, vadimezan carried by platelets containing anti-PD-1 antibodies can activate the immune system to express anti-tumor effects by disrupting blood vessels at tumor sites (172).

## 6 Conclusion

In this review, by analyzing studies reporting macroscopic blood signature changes to microscopic molecular mechanisms, we elucidate the contribution of platelets at different stages of tumor progression and their interactions with immune cells in the TME. Future investigations should further evaluate the underlying mechanism of the interaction between platelets and tumor cells to find new platelet-targeted anti-tumor strategies as adjuvant therapies for surgery. Circulating tumor DNA, CTCs, extracellular vesicles, and TEPs may serve as valuable biomarkers for cancer screening and diagnosis, and further clinical trials are needed to validate the cancer types closely associated with these features. TEP-based liquid biopsy is currently a commonly used diagnostic tool, and its reliability needs to be further demonstrated. Although many experimental studies have identified platelet-dependent tumor metastasis mechanisms, antiplatelet drugs have not been routinely used in clinical settings to prevent and treat tumor metastasis. Experimental validation of different drug-susceptible tumor types is required, and effects on hemostasis and immune responses need to be evaluated to choose treatments that effectively block tumor metastasis with minimal potential adverse effects.

## Author contributions

LW and EG conceptualized the review. LW organized the literature and wrote the manuscript. LW and XW prepared the figures and table. Both SM and XM reviewed and revised the final manuscript.

## Funding

This work was supported by the Postdoctoral Scientific Research Developmental Fund of Heilongjiang Province (LBH-Q18088).

## Conflict of interest

The authors declare that the research was conducted in the absence of any commercial or financial relationships that could be construed as a potential conflict of interest.

## Publisher’s note

All claims expressed in this article are solely those of the authors and do not necessarily represent those of their affiliated organizations, or those of the publisher, the editors and the reviewers. Any product that may be evaluated in this article, or claim that may be made by its manufacturer, is not guaranteed or endorsed by the publisher.

## Abbreviations

ADP, adenosine disphosphate; ATP, adenosine triphosphate; ATX, autotaxin; CAFs, cancer-associated fibroblasts; cAMP, cyclic AMP; CCL, chemokine ligand; CLEC-2, C-type lectin-like receptor-2; COX2, cyclooxygenase2; CTC, circulating tumor cell; CXCL, C-X-C motif ligand; ECM, extracellular matrix; EGF, epidermal growth factor; EGFR, epidermal growth factor receptor; EMT, epithelial-mesenchymal transition; GP, glycoprotein; HT, hydroxytryptamine; H3Cit, citrullinated histone H3; IFN, interferon; IL, interleukin; LPA, lysophosphatidic acid; MMP, matrix metalloproteinase; MPO, myeloperoxidase; NE, norepinephrine; NET, neutrophil extracellular trap; NK, natural killer; NKG2D, Natural-killer group 2 member D; PAR, protease-activated receptor; PCR, polymerase chain reaction; PDGF, platelet-derived growth factor; PD-1, programmed cell death protein 1; PD-L1, programmed death-ligand 1; PGE2, prostaglandin E2; PKA, protein kinase A; PSGL, P-selectin-glycoprotein ligand; PM, platelet membrane; TCIPA, tumor cell-induced platelet aggregation; TEP, tumor-educated platelet; TGF, transforming growth factor; TLR, toll-like receptor; TME, tumor microenvironment; TSP, thrombospondin; TXA2, thromboxane A2; VEGF, vascular endothelial growth factor; 12-HETE, 12-hydroxyeicosatetraenoic acid.
